# Prevalence and Clinical Correlates of Iron Deficiency in Vietnamese Patients with Chronic Heart Failure

**DOI:** 10.3390/biomedicines14040821

**Published:** 2026-04-03

**Authors:** Thanh Van Le, Vinh Thanh Tran, Ai Thi Kim Le, Linh Ha Khanh Duong

**Affiliations:** Department of Biochemistry, Cho Ray Hospital, Ho Chi Minh City 700000, Vietnam; levanthanh09091963@gmail.com (T.V.L.); thanhvinhtran2002@gmail.com (V.T.T.); lethikimai01@gmail.com (A.T.K.L.)

**Keywords:** chronic heart failure, iron deficiency, ferritin, NT-proBNP, C-reactive protein, Vietnam

## Abstract

**Background/Objectives**: Iron deficiency (ID) is a critical comorbidity in chronic heart failure (CHF) that impairs myocardial energy metabolism and clinical outcomes. Despite its significance, data regarding ID prevalence in Southeast Asian CHF populations remain insufficient. This study aimed to determine the prevalence of ID and identify its independent clinical correlates among CHF patients at a leading tertiary hospital in Vietnam using high-precision automated diagnostic platforms. **Methods**: A descriptive cross-sectional study was conducted on 138 adult CHF patients. Serum iron and ferritin were quantified using photometric and electrochemiluminescence immunoassay (ECLIA) methods on cobas c702 and e602 systems. ID was defined according to 2021 ESC guidelines (ferritin < 100 ng/mL or ferritin 100–299 ng/mL with TSAT < 20%). Multivariable logistic regression identified independent factors associated with ID. **Results**: The overall ID prevalence was 75.4%, consisting of functional (39.9%) and absolute ID (35.5%). A significant gender disparity was observed (*p* = 0.0326): absolute ID was more prevalent in females (44.4%), while functional ID predominated in males (47.4%). Multivariable analysis revealed that NT-proBNP (aOR = 2.29; 95% CI: 1.13–4.66; *p* = 0.021) and C-reactive protein (aOR = 2.17; 95% CI: 1.07–4.43; *p* = 0.031) were independent correlates of ID status. The association with anemia was borderline non-significant (*p* = 0.055). **Conclusions**: ID is nearly ubiquitous among Vietnamese CHF patients in this tertiary setting, driven by hemodynamic severity and systemic inflammation. These findings advocate for integrating routine iron status screening into the standard diagnostic workup for all CHF patients, regardless of hemoglobin levels.

## 1. Introduction

Heart failure (HF) represents the terminal stage of various cardiovascular diseases and has emerged as a significant global health challenge [1]. Current epidemiological data indicates that the prevalence of heart failure is approximately 1% to 2% among the adult population in Europe, with a sharp increase to over 10% in individuals aged 70 and older [2,3]. Similarly, Asian countries report a prevalence rate ranging from 1% to 3% [4]. Despite advancements in diagnostic modalities and therapeutic interventions that have improved prognosis in recent years, morbidity and mortality rates associated with HF remain high [1].

In the complex pathophysiology of chronic heart failure (CHF), iron deficiency (ID) is recognized as a critical and frequent comorbidity, present in 30% to 50% of patients [5,6,7]. Iron is a fundamental micronutrient essential for life, supporting the regulation of protein and enzyme activities [8]. Beyond its well-known role in hemoglobin synthesis for oxygen transport, iron is a vital component of myoglobin and enzymes involved in the mitochondrial respiratory chain, DNA synthesis, and immune function. In the context of heart failure, ID impairs myocardial energy metabolism and skeletal muscle function, contributing to reduced exercise capacity, frequent hospitalizations, and an increased risk of cardiovascular mortality, regardless of the presence of anemia [6,9,10].

The scientific community distinguishes between two forms of iron deficiency in CHF: absolute and functional [7,11]. Absolute ID is characterized by the depletion of total body iron stores, whereas functional ID involves a state where iron stores may be sufficient or even increased, but iron mobilization for targeting tissues is impaired due to chronic systemic inflammation [11,12]. This inflammatory state, typical in CHF, triggers the release of cytokines such as IL-6, which stimulates the production of hepcidin [13,14,15]. Elevated hepcidin levels lead to the degradation of ferroportin, thereby inhibiting intestinal iron absorption and sequestering iron within the reticuloendothelial system [6,11,16].

To address this, the European Society of Cardiology (ESC) 2021 and 2023 guidelines recommend systematic and periodic screening for ID in all HF patients using serum ferritin and transferrin saturation (TSAT) [7,17]. The consensus definition for ID in HF is a serum ferritin level < 100 ng/mL (absolute ID), or a ferritin level between 100 and 299 ng/mL if the TSAT is <20% (functional ID) [7,17]. This higher ferritin threshold is necessary because ferritin acts as an acute-phase reactant and can be falsely elevated by the systemic inflammation inherent in heart failure [6].

Global and local studies have highlighted the varying prevalence and impact of ID in different HF populations [18,19,20]. Internationally, the Geneva-based study by Lindberg et al. found an ID prevalence of 54% in patients with heart failure and preserved ejection fraction (HFpEF) [18]. Furthermore, large cohort studies have demonstrated that ID, rather than anemia itself, is independently associated with poor quality of life, as measured by the Minnesota Living with Heart Failure Questionnaire (MLHFQ) [21,22,23,24].

In Vietnam, research regarding iron deficiency in heart failure is gaining traction in the international literature. Recent studies have collectively indicated a high prevalence of ID among Vietnamese patients with chronic HF and reduced ejection fraction [19,20,25]. These investigations have consistently established a correlation between ID and greater disease severity, characterized by higher NYHA functional class, prolonged hospitalizations, and elevated levels of NT-proBNP and hs-CRP [19]. These findings underscore the clinical relevance of iron status in the Vietnamese HF population.

Despite the established importance of iron screening, data from large-scale tertiary centers in Vietnam—where patients often present with advanced disease stages and multiple comorbidities—remains insufficient. Cho Ray Hospital, as a leading special-grade medical institution, manages a diverse and high-volume cohort of CHF patients, making it a critical site for gathering representative clinical data. Accurate assessment of iron status is paramount, requiring highly sensitive and automated laboratory methods.

This study was designed to provide an updated and comprehensive analysis of iron status in CHF patients, utilizing advanced diagnostic platforms. We employed the photometric method on the cobas c702 for serum iron (Fe) determination and the electrochemiluminescence immunoassay (ECLIA) on the cobas e602 for ferritin quantification. These systems provide the precision and wide measuring ranges necessary for reliable clinical research.

The specific objectives of this study are to determine serum iron (Fe) concentrations using the photometric method and ferritin levels via the ECLIA method in patients with chronic heart failure. Furthermore, the research aims to analyze these concentrations to identify the stages of anemia and iron deficiency based on current international standards. By identifying the prevalence and clinical correlates of iron deficiency (ID) in this setting, this study emphasizes the necessity of routine iron screening to optimize the clinical management and quality of life for heart failure patients in Vietnam.

## 2. Materials and Methods

### 2.1. Study Design, Setting, and Participants

This study employed a descriptive cross-sectional design to investigate the prevalence and clinical correlates of ID among patients with CHF. The research was conducted at the Biochemistry Department of Cho Ray Hospital, a premier special-grade tertiary medical center in Ho Chi Minh City, Vietnam, between November 2023 and July 2024. We enrolled adult patients aged ≥18 years who were diagnosed with CHF at the hospital’s Internal Cardiology Department. The diagnosis of CHF was established in accordance with the clinical criteria and diagnostic algorithms of the 2021 European Society of Cardiology (ESC) Guidelines [7] for the diagnosis and treatment of acute and chronic heart failure. Patients were excluded if they presented with concurrent primary hematological disorders (such as acute leukemia, aplastic anemia, or myelodysplastic syndrome), severe liver dysfunction (ALT/AST > 3 times or bilirubin > 2 times the upper limit of normal), or a history of surgery, erythropoietin treatment, or blood transfusion within the preceding three months. Furthermore, individuals currently receiving iron supplementation or anemia treatment within the past 12 months were excluded to ensure an accurate assessment of baseline iron status.

### 2.2. Sample Size and Sampling Procedure

The minimum required sample size was determined using the single proportion formula. Based on a previous study by Dam Hai Son et al. [25], which reported an ID prevalence (*p*) of 41.7% among CHF patients, and setting the margin of error (d) at 0.09 with a 95% confidence level, the minimum sample size was calculated as 115 patients. Utilizing a convenience sampling method, we eventually recruited a total of 138 eligible patients who met all inclusion criteria during the study period. To maintain objectivity and minimize bias, laboratory personnel performing the assays were blinded to the clinical severity and NYHA classification of the participants, and all patient data were de-identified and assigned unique alphanumeric codes for processing (masking/coding).

### 2.3. Clinical Definitions and Criteria

Iron deficiency was categorized into three groups based on the 2021 European Society of Cardiology (ESC) guidelines: (1) Absolute ID, defined as serum ferritin < 100 ng/mL; (2) Functional ID (or relative ID), defined as serum ferritin between 100 and 299 ng/mL with a transferrin saturation (TSAT) < 20%; and (3) No ID, defined as ferritin ≥ 100 ng/mL and TSAT ≥ 20%. Anemia was defined according to World Health Organization (WHO) standards as a hemoglobin concentration < 130 g/L for men and <120 g/L for non-pregnant women. Additionally, nutritional status was assessed via the Body Mass Index (BMI), and dyslipidemia was identified based on standard thresholds for total cholesterol (>200 mg/dL), triglycerides (>150 mg/dL), HDL (<40 mg/dL), and LDL (>100 mg/dL).

### 2.4. Laboratory Procedures and Data Collection

Venous blood samples were collected from participants in the morning following an overnight fast of at least 8 h. Serum was separated within one hour of collection through centrifugation and checked for quality; samples showing hemolysis, turbidity, or inadequate volume were discarded. Serum iron (Fe) was quantified using the photometric method on the cobas c702 system (Roche Diagnostics, Basel, Switzerland), with a measuring range of 0.90–179 µmol/L. Serum ferritin was determined via the electrochemiluminescence immunoassay (ECLIA) on the cobas e602 system, with a measuring range of 0.5–2000 ng/mL. TSAT was calculated using the standard formula: TSAT (%) = [Serum Iron (µmol/L)/(Transferrin (mg/dL))] × 398. Other biochemical markers, including NT-proBNP, high-sensitivity CRP, creatinine, BUN, and complete blood counts, were measured using automated laboratory platforms compliant with ISO 15189:2022 standards [26] to ensure analytical quality.

### 2.5. Statistical Analysis

Data were analyzed using MedCalc software version 24.1.1 (MedCalc Software Ltd., Ostend, Belgium). The normality of quantitative variables was assessed using the Kolmogorov–Smirnov test. Normally distributed data were expressed as mean ± standard deviation (SD), while non-normally distributed data (such as iron, ferritin, and NT-proBNP) were presented as median and interquartile range (IQR). Categorical variables were described as frequencies and percentages. Differences between groups were compared using the Chi-square test for categorical data, the independent *t*-test for normally distributed continuous data, and the Mann–Whitney U test or the Kruskal–Wallis test for non-parametric data. Multivariable logistic regression analysis was performed to identify independent factors associated with ID, calculating adjusted odds ratios (aOR) and 95% confidence intervals (CIs). Variable selection for the multivariable model was performed using Akaike Information Criteria (AIC) minimization to ensure model parsimony and avoid the pitfalls of stepwise selection. Model discrimination was assessed using the c-index, and calibration was verified via the Hosmer–Lemeshow test. A two-tailed *p*-value < 0.05 was considered statistically significant.

### 2.6. Ethical Considerations

The study protocol was reviewed and approved by the Institutional Review Board of Hong Bang International University (Decision No. 1189/QD-HIU, dated 3 November 2023) and the Ethics Committee of Cho Ray Hospital (Decision No. 114/2023/BVTN-HDYD, dated 30 November 2023). All procedures were conducted in accordance with the Declaration of Helsinki. Patient confidentiality was strictly maintained, and the research results were used solely for scientific purposes without incurring any additional costs to the participants beyond standard care.

## 3. Results

### 3.1. Baseline Characteristics of the Study Population

A total of 138 patients diagnosed with CHF were enrolled in the study. The mean age of the study population was 64.76 ± 18.25 years. Demographic analysis showed female predominance (58.7% vs. 41.3%, *p* = 0.0411). Nearly half of the participants (48.6%) were classified as overweight or obese based on the BMI. Laboratory assessments indicated severe cardiac and inflammatory stress, characterized by a median NT-proBNP of 1019.1 pg/mL and a median CRP of 25.0 mg/L. Anemia was highly prevalent, affecting 87.7% of the cohort according to WHO criteria. Detailed baseline characteristics and hematological parameters are summarized in Table 1.

### 3.2. Distribution of Iron Status and Ferritin Profiles

**Figure 1:**
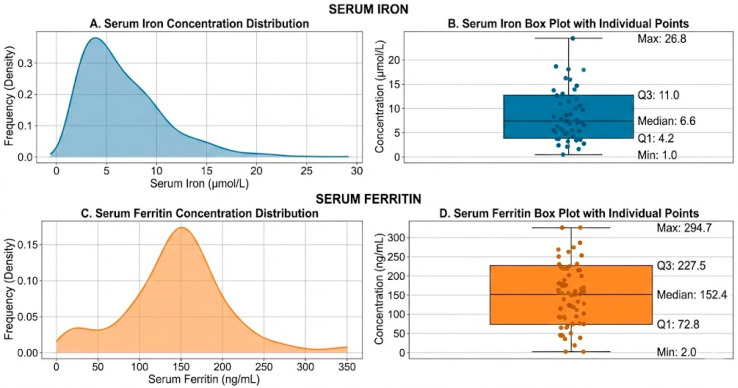
Distribution and Box Plots of Serum Iron and Ferritin Concentrations. (**A**) Density plot illustrating the right-skewed, non-normal distribution of serum iron concentration (μmol/L) in the study population (*n* = 138). (**B**) Vertical box plot of serum iron with individual data points, showing the median (6.6 μmol/L), interquartile range (IQR: 4.2–11.0 μmol/L), minimum, and maximum values. (**C**) Density plot showing the right-skewed, non-normal distribution of serum ferritin concentration (ng/mL) (*n* = 138). (**D**) Vertical box plot of serum ferritin with individual data points, showing the median (152.4 ng/mL), interquartile range (IQR: 72.8–227.5 ng/mL), minimum, and maximum values. Note: Kolmogorov–Smirnov tests confirmed a non-normal distribution for both serum iron and ferritin (*p* < 0.001 for both). The box plots display the 25th percentile (Q1), median, and 75th percentile (Q3), with whiskers extending to the minimum and maximum values.

Serum iron and ferritin levels exhibited non-normal, right-skewed distributions (*p* < 0.001). For the entire cohort, the median serum iron concentration was 6.6 µmol/L (IQR: 4.2–11.0), while the median serum ferritin was 152.4 ng/mL (IQR: 72.8–227.5) (Figure 1).

Subgroup analysis revealed that serum ferritin levels differed significantly by gender, with males showing higher median levels compared to females (166.7 ng/mL vs. 139.7 ng/mL, *p* = 0.031). Additionally, patients with triglycerides > 150 mg/dL had significantly higher ferritin levels (*p* = 0.004), while those with HDL-cholesterol < 40 mg/dL exhibited significantly lower serum iron concentrations (*p* = 0.007) (Table 2).

### 3.3. Prevalence and Phenotypes of Iron Deficiency

Applying the ESC 2021 diagnostic criteria [7], the overall prevalence of ID was 75.4% (*n* = 104). Functional ID was identified as the most frequent phenotype, occurring in 39.9% of patients, followed by absolute ID in 35.5%. A significant gender-based disparity was observed in the distribution of ID types (*p* = 0.032). Absolute ID was the dominant form in females (44.4%), whereas functional ID was more common in males (47.4%). Only 24.6% of the cohort maintained sufficient iron stores (Table 3).

### 3.4. Independent Clinical Correlates of Iron Deficiency

In the univariate logistic regression analysis, ID was significantly associated with three factors:

- Anemia: OR = 4.32 (95% CI: 1.51–12.33; *p* = 0.006).
- NT-proBNP: OR = 2.34 (95% CI: 1.21–4.54; *p* = 0.009).
- CRP: OR = 2.37 (95% CI: 1.25–4.51; *p* = 0.007).

**Figure 2:**
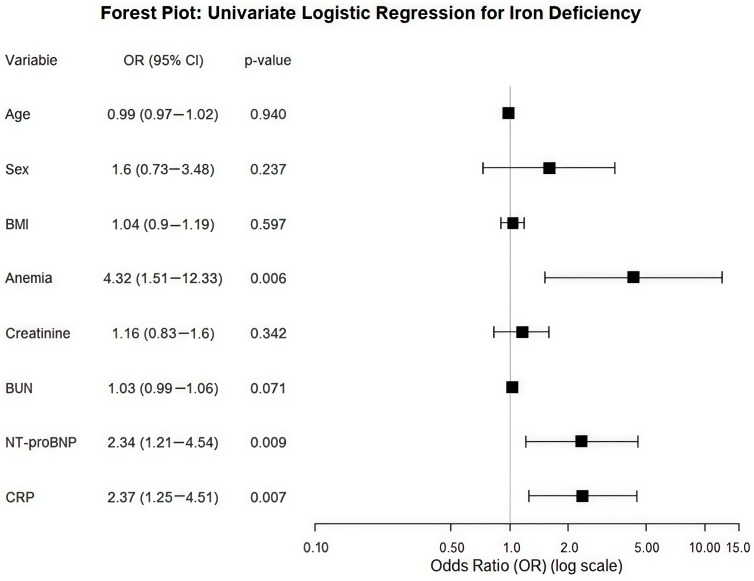
Forest plot of the univariate logistic regression analysis identifying clinical correlates associated with iron deficiency in chronic heart failure patients (N = 138). The figure displays the Odds Ratios (ORs) and 95% Confidence Intervals (CIs) for various clinical and biochemical variables. The dashed vertical line represents the null effect (OR = 1.0). Factors located to the right of the reference line with a CI not crossing 1.0—specifically anemia (OR: 4.32; *p* = 0.006), NT-proBNP (OR: 2.34; *p* = 0.009), and CRP (OR: 2.37; *p* = 0.007)—are significantly associated with an increased likelihood of iron deficiency in this cohort. Variables such as age, sex, and the BMI did not show statistically significant associations in the univariate model (*p* > 0.05).

Variables such as age, gender, the BMI, and renal function (creatinine, BUN) did not demonstrate significant associations (*p* > 0.05) (Figure 2).

**Figure 3:**
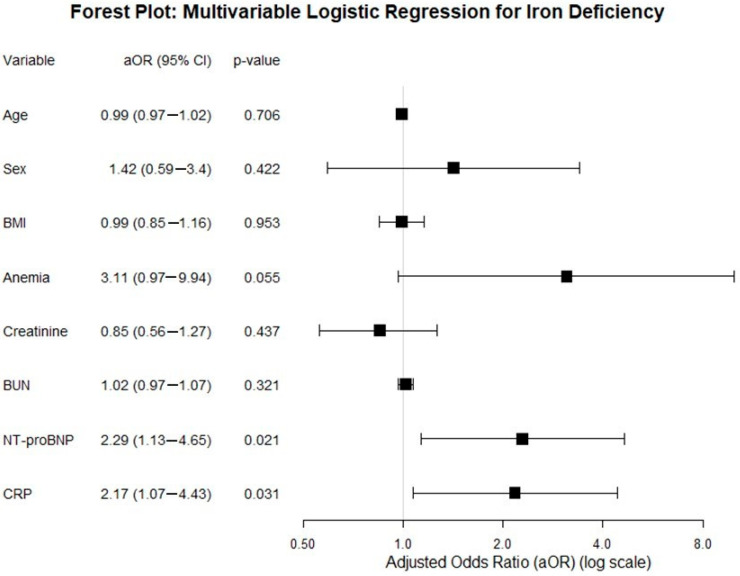
Forest plot of univariate logistic regression analysis for clinical and biochemical factors associated with iron deficiency.

Multivariable logistic regression was subsequently performed to control for confounding variables including age, gender, the BMI, anemia, and renal function. After adjustment, both NT-proBNP (aOR = 2.29; 95% CI: 1.13–4.66; *p* = 0.021) and CRP (aOR = 2.17; 95% CI: 1.07–4.43; *p* = 0.031) remained independent correlates of iron deficiency. The association between ID and anemia became borderline non-significant in the adjusted model (aOR = 3.11; 95% CI: 0.98–9.94; *p* = 0.055) (Figure 3).

The final multivariable model exhibited strong performance, with a C-index of 0.81 and a Hosmer–Lemeshow *p*-value of 0.42, indicating excellent discrimination and calibration.

## 4. Discussion

This study was predicated on the hypothesis that ID remains an underdiagnosed yet pervasive comorbidity among Vietnamese patients with CHF, particularly those managed at tertiary care facilities. Our primary objective was to quantify serum iron and ferritin levels using high-precision automated platforms and to determine the prevalence of ID according to the latest international standards. The findings reveal a strikingly high ID prevalence of 75.4% within our cohort, with functional ID (39.9%) slightly outperforming absolute ID (35.5%) as the dominant phenotype. Furthermore, we identified NT-proBNP and CRP as independent clinical correlates of iron status, suggesting that both hemodynamic severity and systemic inflammation are intrinsically linked to iron metabolic derangements in this population.

When compared to the global and local literature, our observed prevalence of 75.4% significantly exceeds the 30% to 50% range typically reported in European cohorts [18,27,28,29,30] and the 36.5% to 47.8% previously observed in Vietnamese settings [19,20,25]. This discrepancy can be attributed to the “tertiary center effect,” where Cho Ray Hospital manages a high volume of patients with advanced-stage disease and multiple comorbidities. The high median NT-proBNP (1019.1 pg/mL) and CRP (25.0 mg/L) levels in our participants underscore a population with severe cardiac dysfunction and heightened inflammatory activity [11,17]. Notably, our findings regarding gender-specific distributions—where females exhibited a higher rate of absolute ID (44.4%) while males predominantly displayed functional ID (47.4%)—align with the biological understanding that pre-existing low iron stores in women may predispose them to absolute depletion when facing the metabolic demands of heart failure [30]. 

To explain these results, we propose a “Congestive-Inflammatory Metabolic Model” for ID in advanced heart failure. In this model, the severity of heart failure (indicated by high NT-proBNP) leads to chronic gut congestion and reduced nutrient absorption, while concomitant systemic inflammation (indicated by high CRP) triggers the IL-6/hepcidin pathway [9,31,32]. Elevated hepcidin degrades ferroportin, sequestering iron within the reticuloendothelial system and preventing its utilization by mitochondria in the myocardium and skeletal muscles [6,12,33]. This creates a vicious cycle where cardiac stress exacerbates iron sequestration, which in turn impairs myocardial energy metabolism, further worsening cardiac output [11,12,17,28]. The borderline significance of anemia in our multivariable model (*p* = 0.055) suggests that ID is not merely a precursor to anemia but an independent metabolic failure that occurs even before hemoglobin levels drop.

The implications of this study are profound for the management of heart failure in Vietnam. Given that over three-quarters of these patients suffer from ID, the current practice of performing focused screening only for anemic patients is likely missing a vast majority of those who could benefit from iron replacement therapy. Our results advocate for the systematic integration of ferritin and TSAT screening into the standard diagnostic workup for all CHF patients, regardless of their hemoglobin status, to optimize exercise capacity and reduce the risk of cardiovascular mortality.

Despite the robust findings, several limitations must be acknowledged. First, the cross-sectional design precludes the ability to establish a definitive causal relationship between NT-proBNP/CRP and the development of ID over time. Second, the use of convenience sampling at a single tertiary hospital may introduce selection bias, as these patients likely represent a more severe clinical spectrum than those in primary or secondary care. Third, the study did not measure soluble transferrin receptor (sTfR) or hepcidin levels, which could have provided a more nuanced differentiation between absolute and functional ID in the presence of the extremely high CRP levels observed in our cohort. Finally, we did not assess long-term clinical outcomes or the impact of iron supplementation on these specific patients, leaving the therapeutic efficacy in this Vietnamese sub-population to be validated by future longitudinal or interventional trials.

## 5. Conclusions

In conclusion, iron deficiency is a nearly ubiquitous comorbidity in Vietnamese patients with chronic heart failure at the tertiary level, affecting 75.4% of the population. The strong independent associations with NT-proBNP and CRP emphasize that as heart failure progresses and inflammation intensifies, the disruption of iron metabolism becomes more severe. Clinicians should prioritize routine iron screening for all heart failure patients, particularly those with high inflammatory markers or advanced cardiac dysfunction, to bridge the gap between current diagnostic protocols and the actual metabolic needs of patients.

## Figures and Tables

**Table 1:** Baseline Characteristics and Laboratory Parameters of the Study Population.

Characteristics	Total (*n* = 138)
Demographics and Anthropometrics	
Age (years), mean ± SD	64.7 ± 18.2
Gender, *n* (%)	
- Male	57 (41.3)
- Female	81 (58.7)
Body Mass Index (BMI, kg/m^2^), mean ± SD	22.5 ± 2.8
BMI Categories, *n* (%)	
- Underweight (le 18.5)	17 (12.3)
- Normal (18.5–22.9)	54 (39.1)
- Overweight (23.0–24.9)	43 (31.2)
- Obese (≥25.0)	24 (17.4)
Biochemical Parameters, Median (IQR)	
Fasting Glucose (mg/dL)	117.5 (100.5–175.1)
Blood Urea Nitrogen (BUN, mg/dL)	24.0 (16.0–29.1)
Serum Creatinine (mg/dL)	1.1 (0.7–1.5)
NT-proBNP (pg/mL)	1019.1 (398.1–1985.0)
C-Reactive Protein (CRP, mg/L)	25.0 (12.7–59.0)
Lipid Profile, Median (IQR)	
Total Cholesterol (mg/dL)	138.5 (105.1–171.6)
Triglycerides (mg/dL)	115.7 (74.4–162.2)
HDL-Cholesterol (mg/dL)	31.5 (25.7–40.9)
LDL-Cholesterol (mg/dL)	71.8 (50.5–99.1)
Hematological Parameters, Median (IQR)	
Red Blood Cell (RBC, T/L)	3.7 (3.2–4.3)
Hemoglobin (g/L)	92.5 (82.0–112.0)
Hematocrit (%)	29.3 (25.8–34.3)
Mean Corpuscular Volume (MCV, fL)	84.9 (74.3–88.8)
Mean Corpuscular Hemoglobin (MCH, pg)	27.3 (22.9–29.3)
Iron Status Parameters, Median (IQR)	
Serum Iron (µmol/L)	6.6 (4.2–11.0)
Serum Ferritin (ng/mL)	152.4 (72.8–227.5)

**Table 2:** Serum Iron and Ferritin Levels across Demographic and Metabolic Subgroups (N = 138).

Variable	*n*	Serum Iron (μmol/L)	*p*-Value	Serum Ferritin (ng/mL)	*p*-Value
Overall	138	6.6 (4.2–11.0)		152.4 (72.8–227.5)	
Gender			0.209		0.031
Male	57	7.8 (4.7–11.4)		166.7 (102.7–232.7)	
Female	81	6.0 (4.1–10.8)		139.7 (46.8–215.9)	
BMI Category			0.080		0.410
Underweight (≤18.5)	17	7.0 (4.4–9.3)		151.4 (109.3–242.7)	
Normal (18.5–22.9)	54	6.5 (4.2–11.2)		158.2 (74.2–255.5)	
Overweight (23.0–24.9)	43	9.0 (5.1–11.9)		146.2 (73.7–211.9)	
Obese (≥25.0)	24	5.0 (2.6–8.9)		144.3 (57.3–189.9)	
HDL-Cholesterol			0.007		0.958
<40 mg/dL	99	6.0 (4.0–10.2)		149.3 (74.7–231.4)	
≥40 mg/dL	39	9.4 (5.6–13.0)		166.4 (62.7–220.4)	
Triglycerides			0.430		0.004
≤150 mg/dL	96	6.9 (4.2–11.6)		119.1 (56.4–222.8)	
>150 mg/dL	42	6.4 (4.6–10.0)		174.9 (137.8–246.1)	

Notes: Data presented as Median (IQR). *p*-values calculated using the Mann–Whitney U test (for 2 groups) or the Kruskal–Wallis test (for >2 groups).

**Table 3:** Distribution of Iron Deficiency Status by Gender (ESC 2021 Criteria [7]).

ID Status	Total (N = 138)	Male (*n* = 57)	Female (*n* = 81)	*p*-Value
Iron Deficiency	104 (75.4%)	40 (70.2%)	64 (79.0%)	0.032
- Absolute ID	49 (35.5%)	13 (22.8%)	36 (44.4%)	
- Functional ID	55 (39.9%)	27 (47.4%)	28 (34.6%)	
No Iron Deficiency	34 (24.6%)	17 (29.8%)	17 (21.0%)	

Notes: Data presented as *n* (%). Absolute ID: Ferritin < 100 ng/mL. Functional ID: Ferritin 100–299 ng/mL with TSAT < 20%.

## Data Availability

The data presented in this study are available on request from the corresponding author. The data are not publicly available due to hospital privacy regulations and patient confidentiality.

## Abbreviations

The following abbreviations are used in this manuscript:

ALT	Alanine aminotransferase
aOR	Adjusted odds ratio
AST	Aspartate aminotransferase
BMI	Body mass index
BUN	Blood urea nitrogen
CHF	Chronic heart failure
CI	Confidence interval
CRP	C-reactive protein
ECLIA	Electrochemiluminescence immunoassay
ESC	European Society of Cardiology
HDL	High-density lipoprotein
HF	Heart failure
HFpEF	Heart failure with preserved ejection fraction
hs-CRP	High-sensitivity C-reactive protein
ID	Iron deficiency
IQR	Interquartile range
ISO	International Organization for Standardization
LDL	Low-density lipoprotein
MLHFQ	Minnesota Living with Heart Failure Questionnaire
NT-proBNP	N-terminal pro-B-type natriuretic peptide
NYHA	New York Heart Association
SD	Standard deviation
TSAT	Transferrin saturation
WHO	World Health Organization
